# Posttreatment liver function, but not baseline liver function stratifies patient survival after direct-acting antiviral treatment in decompensated cirrhosis with hepatitis C virus

**DOI:** 10.1007/s00535-023-02039-x

**Published:** 2023-10-13

**Authors:** Yuki Tahata, Hayato Hikita, Satoshi Mochida, Nobuyuki Enomoto, Akio Ido, Hidekatsu Kuroda, Daiki Miki, Masayuki Kurosaki, Yoichi Hiasa, Ryotaro Sakamori, Norifumi Kawada, Taro Yamashita, Goki Suda, Hiroshi Yatsuhashi, Hitoshi Yoshiji, Naoya Kato, Taro Takami, Kazuhiko Nakao, Kentaro Matsuura, Yasuhiro Asahina, Yoshito Itoh, Ryosuke Tateishi, Yasunari Nakamoto, Eiji Kakazu, Shuji Terai, Masahito Shimizu, Yoshiyuki Ueno, Norio Akuta, Masanori Miyazaki, Yasutoshi Nozaki, Masayuki Kabayama, Satoshi Sobue, Akihiro Moriuchi, Tomokatsu Miyaki, Takahiro Kodama, Tomohide Tatsumi, Tomomi Yamada, Tetsuo Takehara

**Affiliations:** 1 Department of Gastroenterology and Hepatology, Osaka University Graduate School of Medicine, 2-2 Yamadaoka, Suita, Osaka 565-0871 Japan; 2 Department of Gastroenterology and Hepatology, Saitama Medical University, Saitama, Japan; 3 First Department of Internal Medicine, Faculty of Medicine, University of Yamanashi, Yamanashi, Japan; 4 Digestive and Lifestyle Diseases, Department of Human and Environmental Sciences, Kagoshima University Graduate School of Medicine and Dental Sciences, Kagoshima, Japan; 5 Division of Gastroenterology and Hepatology, Department of Internal Medicine, Iwate Medical University, Iwate, Japan; 6 Department of Gastroenterology and Metabolism, Graduate School of Biomedical and Health Sciences, Hiroshima University, Hiroshima, Japan; 7 Department of Gastroenterology and Hepatology, Musashino Red Cross Hospital, Tokyo, Japan; 8 Department of Gastroenterology and Metabology, Ehime University Graduate School of Medicine, Ehime, Japan; 9 Department of Hepatology, Graduate School of Medicine, Osaka Metropolitan University, Osaka, Japan; 10 Department of Gastroenterology, Kanazawa University Graduate School of Medicine, Kanazawa, Japan; 11 Department of Gastroenterology and Hepatology, Graduate School of Medicine, Hokkaido University, Sapporo, Hokkaido Japan; 12 Clinical Research Center, National Hospital Organization Nagasaki Medical Center, Nagasaki, Japan; 13 Department of Gastroenterology, Nara Medical University, Nara, Japan; 14 Department of Gastroenterology, Graduate School of Medicine, Chiba University, Chiba, Japan; 15 Department of Gastroenterology and Hepatology, Yamaguchi University Graduate School of Medicine, Yamaguchi, Ube Japan; 16 Department of Gastroenterology and Hepatology, Nagasaki University of Graduate School of Biomedical Sciences, Nagasaki, Japan; 17 Department of Gastroenterology and Metabolism, Nagoya City University Graduate School of Medical Sciences, Nagoya, Japan; 18 Department of Gastroenterology and Hepatology, Department of Liver Disease Control, Tokyo Medical and Dental University, Tokyo, Japan; 19 Department of Molecular Gastroenterology and Hepatology, Graduate School of Medical Science, Kyoto Prefectural University of Medicine, Kyoto, Japan; 20 Department of Gastroenterology, Graduate School of Medicine, The University of Tokyo, Tokyo, Japan; 21 Second Department of Internal Medicine, Faculty of Medical Sciences, University of Fukui, Fukui, Japan; 22 The Research Center for Hepatitis and Immunology, National Center for Global Health and Medicine, Chiba, Japan; 23 Division of Gastroenterology and Hepatology, Graduate School of Medicine and Dental Sciences, Niigata University, Niigata, Japan; 24 Department of Gastroenterology/Internal Medicine, Gifu University Graduate School of Medicine, Gifu, Japan; 25 Department of Gastroenterology, Faculty of Medicine, Yamagata University, Yamagata, Japan; 26 Department of Hepatology, Toranomon Hospital, Tokyo, Japan; 27 Department of Gastroenterology, Osaka Police Hospital, Osaka, Japan; 28 Department of Gastroenterology, Kansai Rosai Hospital, Hyogo, Japan; 29 Department of Gastroenterology, Kagoshima Prefectural Oshima Hospital, Kagoshima, Japan; 30 Department of Gastroenterology, Kasugai Municipal Hospital, Kasugai, Japan; 31 Department of Gastroenterology, National Hospital Organization Kagoshima Medical Center, Kagoshima, Japan; 32 Department of Gastroenterology, Toyokawa City Hospital, Toyokawa, Japan; 33 Department of Medical Innovation, Osaka University Hospital, Suita, Osaka Japan

**Keywords:** DAA, Sofosbuvir, Velpatasvir, Prognosis, Child–Pugh score

## Abstract

**Background:**

The prognosis of cirrhosis is clearly stratified by liver function. Although direct-acting antiviral (DAA) has recently been used to eliminate hepatitis C virus (HCV), it is not clear whether liver function stratifies the prognosis of decompensated cirrhotic patients treated with DAA.

**Methods:**

A total of 206 HCV-associated decompensated cirrhotic patients who started DAA from February 2019 to December 2021 at 31 Japanese hospitals were prospectively registered.

**Results:**

The median age was 68, and the proportions of patients with Child–Pugh class A (CP-A), CP-B and CP-C were 10% (20/206), 76% (156/206) and 15% (30/206), respectively. Twenty-six patients died, and two patients underwent liver transplantation (LT); the 2- and 3-year LT-free survival rates were 90.0% and 83.2%, respectively. We examined factors associated with LT-free survival using 2 models including either CP class (Model 1) or MELD score (Model 2). In multivariate Cox proportional hazard analysis, CP class at 12 weeks after the end of treatment (EOT) in Model 1 and MELD score at 12 weeks after the EOT in Model 2 were significant factors, while baseline CP class or MELD score was not. Two-year LT-free survival rates were 100%, 91.6% and 60.4% for patients with CP-A, CP-B and CP-C at 12 weeks after the EOT and 95.2% and 69.6% for patients with MELD < 15 and MELD ≥ 15 at 12 weeks after the EOT, respectively.

**Conclusions:**

The prognosis of decompensated cirrhotic patients receiving DAA was stratified by liver function at 12 weeks after the EOT, not by baseline liver function.

**Supplementary Information:**

The online version contains supplementary material available at 10.1007/s00535-023-02039-x.

## Introduction

The prognosis of patients with liver cirrhosis is clearly stratified by liver function [1, 2]. A recent Japanese study that included 444 cirrhotic patients with various etiologies reported that the 3- and 5-year survival rates were 74.4% and 61.1% for patients with Child–Pugh class A (CP-A), 58.5% and 42.4% for those with CP-B and 37.3% and 24.9% for those with CP-C, respectively [2]. Even in studies limited to hepatitis C virus (HCV)-associated decompensated cirrhosis, survival rates significantly decreased according to liver function [3–5]. Planas et al. reported that the 5-year survival rates were 69.6%, 46.3% and 36.4% for patients with CP-A, CP-B and CP-C, respectively [3]. Thus, liver function is a strong prognostic factor for patients with liver cirrhosis.

With the recent introduction of direct-acting antiviral (DAA) treatment, HCV can be eliminated in approximately 90% of patients with decompensated cirrhosis. HCV elimination improves liver function in the short term even in patients with decompensated cirrhosis [6–11]. However, it is not clear how strongly baseline liver function determines the prognosis of decompensated cirrhotic patients treated with DAA.

The aim of this study was to evaluate the impact of baseline liver function on the prognosis of patients with decompensated cirrhosis who underwent DAA treatment.

## Methods

### Study patients

This multicentre observational study was performed at Osaka University Hospital and 30 other hospitals in Japan. A total of 206 consecutive HCV-associated decompensated cirrhotic patients who initiated DAA treatment between February 2019 and December 2021 at each hospital were prospectively registered. No exclusion criteria were set. Decompensated cirrhosis was defined as CP-B or CP-C or CP-A with previous decompensating events according to our previous reports [6, 12].

The institutional review board (IRB) at each participating hospital approved the protocol of this study (IRB No. 18431), and this study was performed in accordance with the Declaration of Helsinki as amended in 2013. In principle, informed consent was obtained from participating patients through an opt-out method. We registered this study with the University Hospital Medical Information Network (UMIN36150).

### Antiviral treatment and virologic response

All patients were treated with sofosbuvir plus velpatasvir (SOF/VEL) for 12 weeks according to the HCV guidelines in Japan [13]. Sustained virologic response (SVR) was defined as undetectable serum HCV RNA at 12 or 24 weeks after the end of treatment (EOT).

### Data collection and management

Patients were seen at each hospital every 3 to 6 months after SOF/VEL treatment according to Japanese guidelines for liver cirrhosis [14, 15]. Clinical data such as laboratory tests, liver imaging examinations and physical examinations were collected at the start of treatment, EOT, 12 weeks after the EOT, 24 weeks after the EOT and every 6 months thereafter. Liver imaging examinations such as ultrasonography, computed tomography or magnetic resonance imaging were performed at each hospital before treatment to confirm the absence of hepatocellular carcinoma (HCC). We collected the clinical information of participating patients from electronic medical records, and these data were registered and managed in the Research Electronic Data Capture system [16, 17].

### Liver function

We evaluated CP class and the model for end-stage liver disease (MELD) score as indices of liver function. For the evaluation of CP class, the international normalization ratio (INR) score for patients taking warfarin was set at 1. The ascites score for patients taking diuretics with no ascites by liver imaging examination was set at 2. The hepatic encephalopathy (HE) score for patients taking branched-chain amino acid (BCAA) supplements or anti-hepatic encephalopathy drugs with no symptoms of HE was determined by the attending physician. The MELD score was calculated as 3.78 × ln total bilirubin [mg/dl] + 9.57 × ln creatinine [mg/dl] + 11.20 × ln INR + 6.43 according to previous reports [18–21].

## Statistical analysis

Categorical variables are presented as frequencies, and continuous variables are presented as medians and interquartile ranges. For the survival analysis, the observation period started from the date when DAA treatment was initiated, and the last observation period was defined as the date of death or liver transplantation (LT) or last hospital visit, whichever came first. LT-free survival rates were estimated by Kaplan–Meier curves, and log-rank tests were used to analyze the differences in LT-free survival rates. We examined factors associated with LT-free survival by a Cox proportional hazards model excluding nine patients who died or were lost to follow-up by 12 weeks after the EOT. Factors identified as significant in the univariate model were analyzed in the multivariate model. In these analyses, we used two models that included either CP class (Model 1) or MELD score (Model 2). Factors associated with patients with CP-C at 12 weeks after the EOT were analyzed by a logistic regression model. All statistical analyses were performed using SPSS version 24.0 (IBM, Armonk, NY, USA). A two-tailed p-value less than 0.05 was defined as statistically significant. In the analyses of LT-free survival according to CP class, we used the Bonferroni method for multiple comparisons.

## Results

### Patient characteristics

The median age was 68 years, and 52% of patients were male (Table 1). The distributions of patients with CP-A, CP-B and CP-C were 10% (20/206), 76% (156/206) and 15% (30/206), respectively, and the median MELD score was 11. Six patients had virological relapse, one had no-response, and two were missing HCV RNA data. Five patients died, and four were lost to follow-up before the SVR could be determined. The SVR rate was 91.3% (188/206) in intention-to-treat fashion.

**Table 1:** Patient characteristics

Factor	*N* = 206	Missing
Age (years)	68 (59–76)	0
Sex: Male/female	106/100	0
BMI (kg/m^2^)	24.0 (21.3–26.3)	1
Diabetes mellitus: Yes/no	51/155	0
Alcohol intake (g/day): 0/ < 20/20–59/ ≥ 60	125/25/25/25	6
Genotype: 1/2/3/1 + 2	131/71/1/1	2
Antiviral treatment: Naïve/IFN-based/IFN-free	161/37/8	0
Child–Pugh score: 5/6/7/8/9/10/11/12/13	5/15/75/50/31/19/7/3/1	0
Esophagogastric varix: Absent/F1/F2 or more/history of rupture	46/74/41/17	28
History of HCC treatment: Yes/no	82/124	0
HCV-RNA (log_10_ IU/ml)	5.7 (5.1–6.1)	0
Platelet count (× 10^4^/μl)	8.3 (5.6–11.3)	0
Total bilirubin (mg/dl)	1.3 (0.8–1.9)	0
AST (U/l)	54 (40–75)	0
ALT (U/l)	35 (25–57)	0
Creatinine (mg/dl)	0.8 (0.7–1.0)	0
Albumin (g/dl)	3.0 (2.7–3.4)	0
INR^a^	1.21 (1.12–1.31)	17
FIB-4 index	7.83 (5.38–11.87)	0
MELD score^a^	11 (9–12)	17
Ascites: No/mild/severe	72/110/24	0
Encephalopathy: No/mild/severe	173/33/0	0
AFP (ng/ml)	8.6 (4.4–20.3)	1

^a^The INRs and MELD scores were missing for 17 patients due to the use of warfarin

Continuous variables were presented as medians and interquartile ranges

*BMI* body mass index, *IFN* interferon, *HCC* hepatocellular carcinoma, *HCV* hepatitis C virus, *AST* aspartate aminotransferase, *ALT* alanine aminotransferase, *INR* international normalization ratio, *FIB-4 index* fibrosis-4 index, *MELD* score; model for end-stage liver disease score, *AFP* alpha-fetoprotein

### LT-free survival after SOF/VEL treatment

**Fig. 1:**
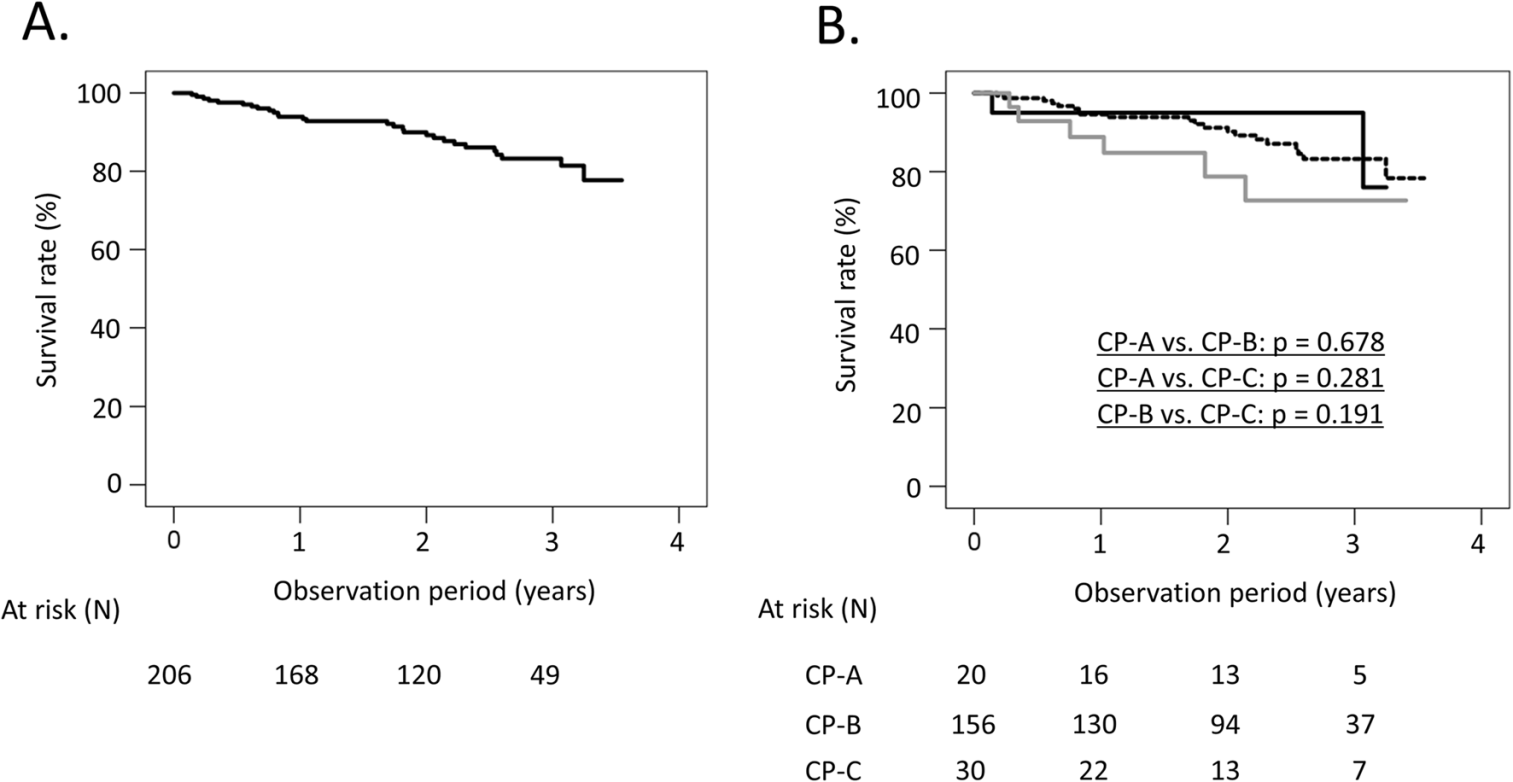
LT-free survival rates among all patients. **A** LT-free survival rates, **B** LT-free survival rates according to Child–Pugh class at baseline. Black solid line, patients with Child–Pugh class A; black dotted line, patients with Child–Pugh class B; grey solid line, patients with Child–Pugh class C. The log-rank test and Kaplan–Meier estimation were used to analyze the differences in LT-free survival rates. We compared survival rates according to CP class by the Bonferroni method for multiple comparisons, and unadjusted *p*-values were shown

Among 206 patients, 26 patients died and two patients underwent LT during the median observation period of 28.1 months from the start of SOF/VEL treatment. The causes of death were liver failure in 13 patients, HCC in four patients, varix rupture, sepsis, heart failure, pancreatic cancer, cerebral infarction, gastric neuroendocrine carcinoma, and cancer of unknown primary in one patient each, and unknown causes in two patients. Two patients underwent LT due to liver failure without HCC. One was a 59-year-old female whose baseline CP score was 9. She achieved SVR, but her CP score deteriorated to 10 at 24 weeks after the EOT, and she underwent LT on day 298 after the start of treatment. The other was a 56-year-old male whose baseline CP score was 9. He achieved SVR, but his CP score deteriorated to 11 at 2 years after the EOT, and he underwent LT on day 833 after the start of treatment. The LT-free survival rates at 2 and 3 years were 90.0% and 83.2%, respectively (Fig. 1A). We compared survival rates according to baseline CP class by the Bonferroni method for multiple comparisons, and unadjusted p-values were shown. The 2 and 3-year LT-free survival rates were 95.0% and 95.0% for patients with CP-A, 91.2% and 83.2% for those with CP-B and 78.7% and 72.7% for those with CP-C, respectively, and there were no significant differences in LT-free survival rates (CP-A vs. CP-B; *p* = 0.678, CP-A vs. CP-C; *p* = 0.281, CP-B vs. CP-C; *p* = 0.191) (Fig. 1B).

### Factors associated with LT-free survival among decompensated cirrhotic patients

We examined factors associated with LT-free survival by Cox proportional hazards model excluding nine patients who died or were lost to follow-up by 12 weeks after the EOT **(**Table 2). The characteristic of patients who died or were lost to follow-up by 12 weeks after the EOT are shown (Supplementary Table 1). In the LT-free survival analysis, 21 patients died and two patients underwent LT. In univariate analysis, the presence of ascites, HE, alanine aminotransferase (ALT) levels, creatinine levels, CP class at 12 weeks after the EOT and MELD score at 12 weeks after the EOT were significant factors. Multivariate analyses were conducted using two models that included either CP class (Model 1) or MELD score (Model 2). We excluded the factors of ascites and HE in the multivariate analysis using Model 1 and creatinine levels in the multivariate analysis using Model 2 since these factors were included in the formula for the CP score or MELD score. In Model 1, ALT levels (hazard ratio (HR): 0.976, 95% confidence interval (CI): 0.952–1.000, *p* = 0.046), creatinine levels (HR: 6.853, 95% CI: 1.964–23.912, *p* = 0.003) and CP-C at 12 weeks after the EOT (HR: 7.931, 95% CI: 2.441–25.773, *p* = 0.001) were significant factors for LT-free survival in multivariate analysis. In Model 2, the presence of HE (HR: 3.051, 95% CI: 1.098–8.474, *p* = 0.032) and MELD score at 12 weeks after the EOT (HR: 1.166, 95% CI: 1.042–1.306, *p* = 0.007) were significant factors for LT-free survival in multivariate analysis.

**Table 2:** Factors associated with LT-free survival after DAA treatment

Factor	Category	Univariate analysis		Multivariate analysis (Model 1)		Multivariate analysis (Model 2)	
		HR (95% CI)	*p* value	HR (95% CI)	*p* value	HR (95% CI)	*p* value
Age	Per 1 year	1.035 (0.994–1.078)	0.094				
Sex	Male/Female	1.371 (0.601–3.131)	0.453				
BMI	Per 1 kg/m^2^	0.938 (0.840–1.049)	0.263				
Diabetes mellitus	Yes/No	1.715 (0.726–4.047)	0.219				
Alcohol intake	Yes/No	1.530 (0.670–3.496)	0.313				
Varix ^a^	Yes/No	2.623 (0.599–11.486)	0.200				
History of HCC	Yes/No	1.560 (0.687–3.540)	0.288				
Ascites	Mild or severe/No	3.573 (1.062–12.028)	0.040			2.287 (0.652–8.021)	0.196
Encephalopathy	Mild or severe/No	2.778 (1.139–6.778)	0.025			3.051 (1.098–8.474)	0.032
Platelet count	Per 1 × 10^4^/μl	0.999 (0.923–1.081)	0.981				
ALT	Per 1 U/l	0.976 (0.954–0.999)	0.040	0.976 (0.952–1.000)	0.046	0.977 (0.952–1.001)	0.061
Total bilirubin	Per 1 mg/dl	1.168 (0.885–1.541)	0.272				
INR	Per 1	0.248 (0.010–6.105)	0.393				
Albumin	Per 1 g/d	1.391 (0.581–3.328)	0.458				
Creatinine	Per 1 g/dl	6.825 (2.108–22.100)	0.001	6.853 (1.964–23.912)	0.003		
Virologic response	Yes/No	1.861 (0.250–13.873)	0.545				
Child–Pugh class at baseline	CP-A	1					
	CP-B	2.483 (0.331–18.615)	0.376				
	CP-C	3.391 (0.378–30.407)	0.275				
Child–Pugh class at 12 weeks after the EOT	CP-A	1		1			
	CP-B	1.782 (0.619–5.132)	0.284	1.323 (0.450–3.892)	0.611		
	CP-C	8.425 (2.666–26.622)	< 0.001	7.931 (2.441–25.773)	0.001		
MELD score at baseline	Per 1	1.102 (0.944–1.286)	0.218				
MELD score at 12 weeks after the EOT	Per 1	1.143 (1.034–1.264)	0.009			1.166 (1.042–1.306)	0.007

a: Patients with an esophagogastric varix of F1 or more at baseline or a history of varix rupture were classified as yes

*DAA* direct-acting antiviral, *HR* hazard ratio, *CI* confidence interval, *BMI* body mass index, HCC; hepatocellular carcinoma, ALT; alanine aminotransferase, *INR* international normalization ratio, *MELD score*; model for end-stage liver disease score, EOT; end of treatment

### LT-free survival rates according to baseline and posttreatment liver function

**Fig. 2:**
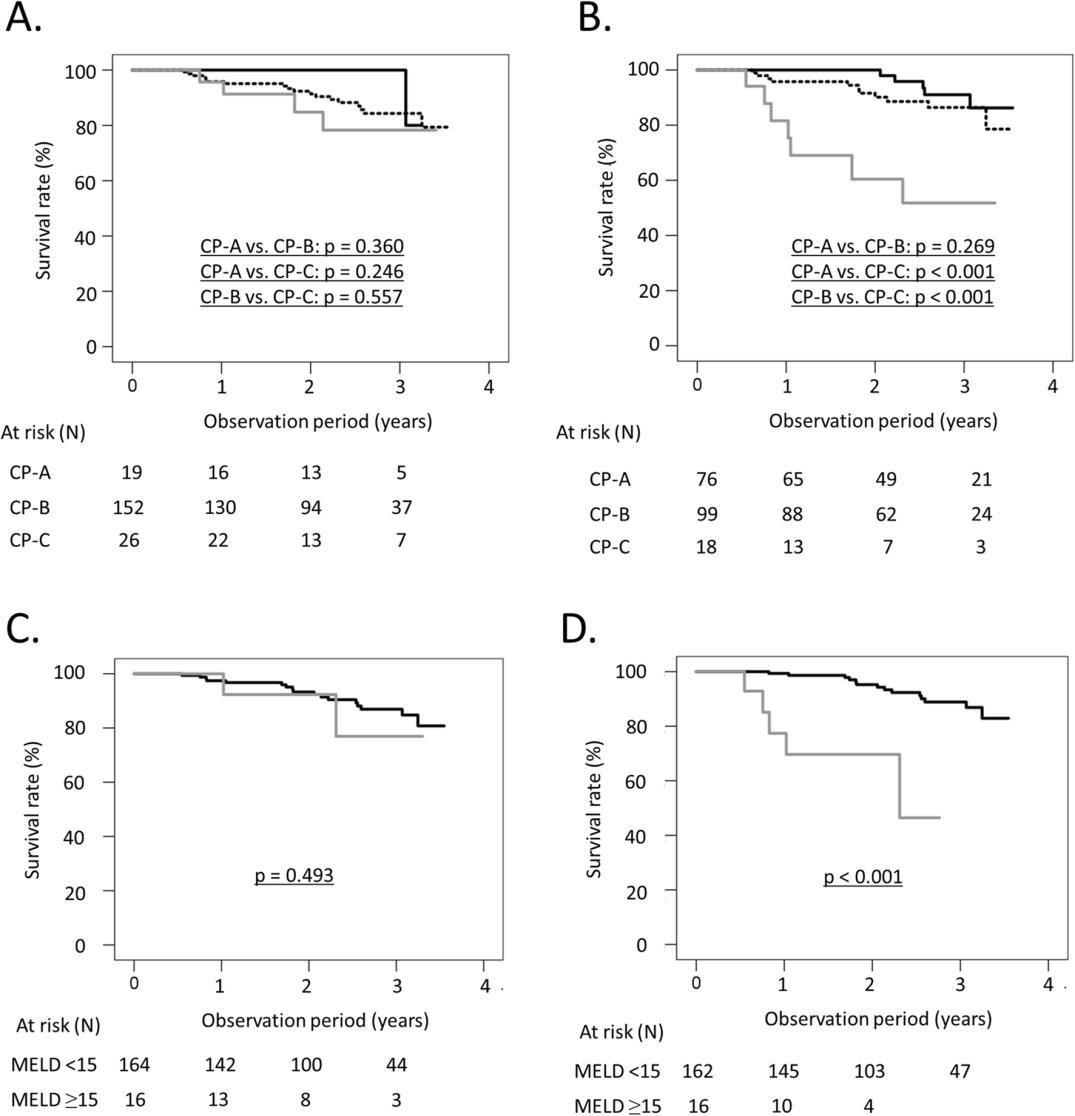
LT-free survival rates excluding nine patients who died or were lost to follow-up by 12 weeks after the EOT. **A**. LT-free survival rates according to Child–Pugh class at baseline. Black solid line, patients with Child–Pugh class A; black dotted line, patients with Child–Pugh class B; grey solid line, patients with Child–Pugh class C. **B**. LT-free survival rates according to Child–Pugh class at 12 weeks after the EOT. Black solid line, patients with Child–Pugh class A; black dotted line, patients with Child–Pugh class B; grey solid line, patients with Child–Pugh class C. **C** LT-free survival rates according to MELD score at baseline. Black line, patients with MELD scores less than 15; grey line, patients with MELD scores of 15 or more. **D** LT-free survival rates according to MELD score at 12 weeks after the EOT. Black line, patients with MELD scores less than 15; grey line, patients with MELD scores of 15 or more. The log-rank test and Kaplan–Meier estimation were used to analyze the differences in LT-free survival rates. We compared survival rates according to CP class by the Bonferroni method for multiple comparisons, and unadjusted p-values were shown. Abbreviations: *EOT* end of treatment; *MELD* score, model for end-stage liver disease score

We examined LT-free survival rates according to CP class or MELD score excluding nine patients who died or were lost to follow-up by 12 weeks after the EOT (Fig. 2). We compared survival rates according to CP class by the Bonferroni method for multiple comparisons, and unadjusted p-values were shown. For baseline CP class, 2- and 3-year LT-free survival rates were 100% and 100% for patients with CP-A, 92.4% and 84.3% for those with CP-B and 84.8% and 78.3% for those with CP-C, respectively, and there were no significant differences in LT-free survival rates (CP-A vs. CP-B; *p* = 0.360, CP-A vs. CP-C; *p* = 0.246, CP-B vs. CP-C; *p* = 0.557) (Fig. 2A). Regarding CP class at 12 weeks after the EOT, the 2- and 3-year LT-free survival rates were 100% and 91.0% for patients with CP-A, 91.6% and 86.4% for those with CP-B and 60.4% and 51.8% for those with CP-C, respectively, and the survival rates of patients with CP-C were significantly lower than those of patients with CP-A or CP-B (CP-A vs. CP-B; *p* = 0.269, CP-A vs. CP-C; *p* < 0.001, CP-B vs. CP-C; *p* < 0.001) (Fig. 2B). Receiver operating characteristic analysis was used to determine the optimal cut-off value of the MELD score at 12 weeks after the EOT for predicting LT-free survival. The optimal cut-off value of the MELD score at 12 weeks after the EOT was 15 by the Youden index. For baseline MELD score, the 2-year LT-free survival rates were 93.3% and 92.3% for patients with MELD scores less than 15 and MELD scores of 15 or more, respectively, and there were no significant differences in LT-free survival rates (*p* = 0.493) (Fig. 2C). Regarding the MELD score at 12 weeks after the EOT, the 2-year LT-free survival rates were 95.2% and 69.6% for patients with MELD scores less than 15 and MELD scores of 15 or more, respectively, and the survival rates of patients with MELD scores of 15 or more were significantly lower than those of patients with MELD scores less than 15 (*p* < 0.001) (Fig. 2D).

### Changes in liver function between baseline and 12 weeks after the EOT

**Fig. 3:**
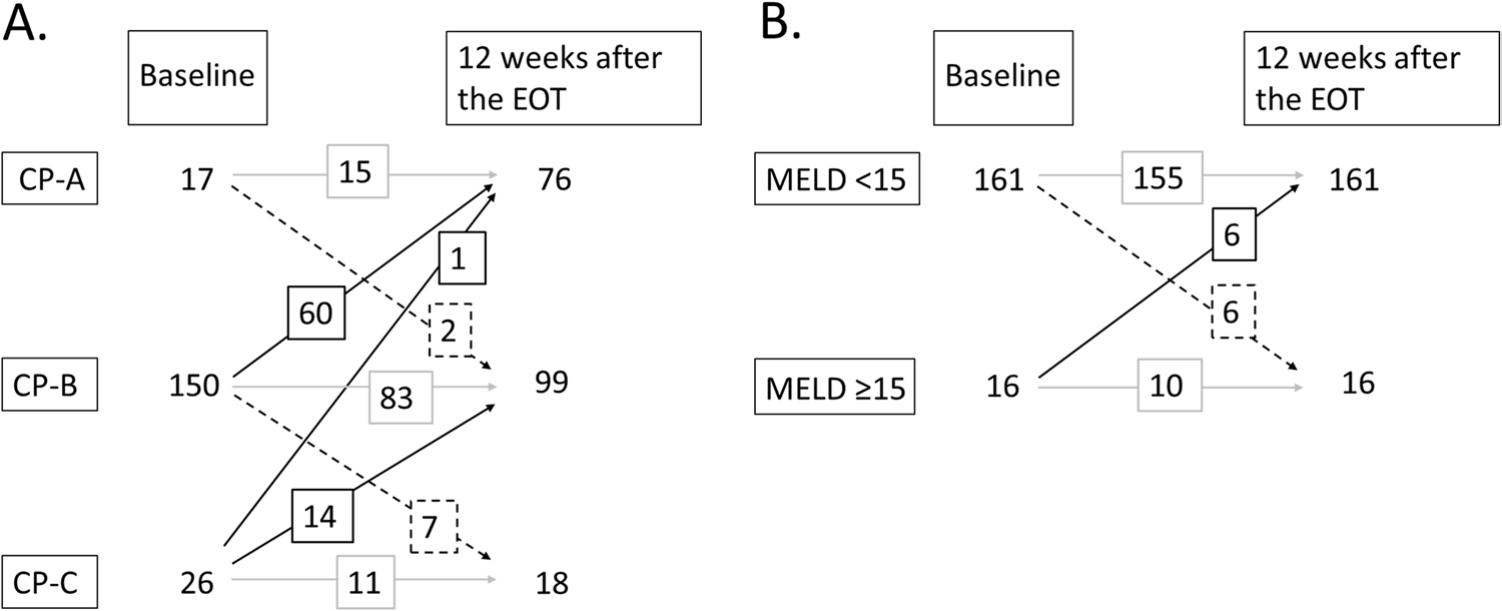
Changes in liver function between baseline and 12 weeks after the EOT. **A**. Child–Pugh class. **B**. MELD score. Black solid line, improvement; grey solid line, stable; black dotted line, worsening. Abbreviations: *EOT* end of treatment; *MELD* score, model for end-stage liver disease score

We examined the changes in CP class or MELD score between baseline and 12 weeks after the EOT (Fig. 3). For CP class, we evaluated CP class both at baseline and 12 weeks after the EOT for 193 patients. Among 150 patients with baseline CP-B, 60 improved to CP-A, 83 remained in CP-B, and seven deteriorated to CP-C at 12 weeks after the EOT. Among 26 patients with baseline CP-C, one and 14 improved to CP-A and CP-B, respectively, and 11 remained in CP-C at 12 weeks after the EOT (Fig. 3A).

For the MELD score, we evaluated the MELD score both at baseline and 12 weeks after the EOT for 177 patients. Among 16 patients with a baseline MELD score of 15 or more, 6 improved to a MELD score less than 15, and 10 maintained a MELD score of 15 or more at 12 weeks after the EOT (Fig. 3B).

### Baseline factors associated with Child–Pugh class C at 12 weeks after the EOT among patients with Child–Pugh class B or C at baseline

Since no patients with CP-A at baseline deteriorated to CP-C at 12 weeks after the EOT, we examined baseline factors associated with CP class C at 12 weeks after the EOT among patients with CP-B or CP-C at baseline by a logistic regression model (Table 3). In the univariate analysis, the presence of HE, total bilirubin levels, the INR and albumin levels were significant factors. In the multivariate analysis, the presence of HE (HR: 4.200, 95% CI: 1.252–14.086, *p* = 0.020) and total bilirubin levels (HR: 1.786, 95% CI: 1.238–2.579, *p* = 0.002) were significant factors (Table 3).

**Table 3:** Baseline factors associated with Child–Pugh class C at 12 weeks after EOT among patients with Child–Pugh class B or C at baseline

		Univariate analysis		Multivariate analysis	
Factor	Category	OR (95% CI)	*p*-value	OR (95% CI)	*p*-value
Age	Per 1 year	0.966 (0.925–1.009)	0.117		
Sex	Male/Female	1.047 (0.392–2.791)	0.928		
BMI	Per 1 kg/m^2^	1.012 (0.891–1.150)	0.853		
Diabetes mellitus	Yes/No	0.571 (0.157–2.073)	0.394		
Alcohol intake	Yes/No	1.411 (0.526–3.785)	0.494		
Varix^a^	Yes/No	0.971 (0.294–3.212)	0.962		
History of HCC	Yes/No	0.774 (0.276–2.170)	0.626		
Ascites	Mild or severe/No	3.925 (0.870–17.711)	0.075		
Encephalopathy	Mild or severe/No	3.735 (1.313–10.628)	0.014	4.200 (1.252–14.086)	0.020
Platelet count	Per 1 × 10^4^/μl	0.898 (0.785–1.029)	0.121		
ALT	Per 1 U/l	0.993 (0.975–1.011)	0.462		
Total bilirubin	Per 1 mg/dl	1.778 (1.258–2.513)	0.001	1.786 (1.238–2.579)	0.002
INR	Per 1	14.230 (1.755–115.391)	0.013	2.950 (0.253–34.384)	0.388
Albumin	Per 1 g/dl	0.209 (0.066–0.666)	0.008	0.279 (0.073–1.070)	0.063
Creatinine	Per 1 g/dl	1.720 (0.333–8.881)	0.517		

^a^Patients with an esophagogastric varix of F1 or more at baseline or a history of varix rupture were classified as yes

*EOT* end of treatment, *OR* odds ratio, *CI* confidence interval, *BMI* body mass index, *HCC* hepatocellular carcinoma, *ALT* alanine aminotransferase, *INR* international normalization ratio, *MELD* score; model for end-stage liver disease score

## Discussion

This study examined whether liver function stratified the prognosis of patients with HCV-associated decompensated cirrhosis who underwent DAA treatment using a prospectively registered cohort. This study revealed that posttreatment liver function, but not baseline liver function, could stratify patients’ survival after DAA treatment.

Liver function has been reported to be useful in stratifying the natural history of patients with cirrhosis [1, 2]. In the present study, CP class and MELD score at 12 weeks after the EOT were significantly associated with LT-free survival after DAA treatment (Table 2). The survival rates of patients with posttreatment CP-C were significantly lower than those of patients with posttreatment CP-A or CP-B, and the survival rates of patients with posttreatment MELD scores of 15 or more were significantly lower than those of patients with posttreatment MELD scores less than 15 (Fig. 2B, D). On the other hand, baseline CP class and MELD score were not significant (Fig. 2A, C). Few studies have reported an association between posttreatment liver function and prognosis among decompensated cirrhotic patients treated with DAA. Pereira et al. reported that the posttreatment MELD score was significantly associated with LT-free survival among decompensated cirrhotic patients who achieved an SVR after DAA treatment [22]. Improvement in liver function after DAA treatment is expected even among decompensated cirrhotic patients, and the posttreatment liver function rather than baseline liver function may be useful for stratifying patient survival. In the present study, creatinine level was extracted as a significant factor independent of CP class in Model 1, while HE was extracted as a significant factor independent of MELD score in Model 2. Considering that creatinine level is included in MELD score and that HE is included in CP class, both CP class and MELD score would be useful prognostic scores for patients with decompensated cirrhosis.

In the present study, the 3-year LT-free survival rate among patients with CP-C at 12 weeks after the EOT was significantly lower than that among patients with CP-A or CP-B (Fig. 2B). Recently, the BE3A score, which consists of body mass index (BMI), HE, ascites, ALT levels and albumin levels, was reported to be useful for predicting patients whose CP class improved to CP-A among patients with decompensated cirrhosis treated with DAA [23]. However, not becoming to CP-C might be more important considering patient prognosis. In the present study, the presence of HE and total bilirubin levels were significant factors associated with patients with CP-C at 12 weeks after the EOT (Table 3). HE is an abnormal neurological or psychiatric manifestation of acute or chronic liver disease, and one of the causes of HE is the presence of a portosystemic shunt [24]. Takaoka et al. reported that baseline total bilirubin levels and the diameter of portosystemic shunts were associated with non-improvement of CP score among decompensated cirrhotic patients who received DAA treatment [7]. Decompensated cirrhotic patients with HE may have an unfavorable prognosis due to poor improvement in CP class after DAA treatment. Future studies are needed to determine which patients will be in CP-C after DAA treatment.

In the present study, LT-free survival rates at 2 and 3 years were 90.0% and 83.2%, respectively, among decompensated cirrhotic patients treated with DAA during an observation period of 28.1 months (Fig. 1). In a European study, Krassenburg et al. [25] reported the prognosis of 149 patients with decompensated cirrhosis treated with DAA. In this report, the median age of the patients was 59 years, and the proportions of patients with CP-B and CP-C were 85% and 15%, respectively. During 27 months, 19 patients underwent LT, and the LT-free survival rate at 2 years was 81.2%. In another European study, Pageaux et al. [26] reported the prognosis of 483 patients with decompensated cirrhosis treated with DAA. In this report, the median age of the patients was 56.6 years, and the proportions of patients with CP-A, CP-B and CP-C were 57%, 37% and 6%, respectively. During 43.5 months, 26 patients underwent LT, and all-cause mortality was 5.2 per 100 patient-years. In the present study, the median age of the patients was 68 years, only two patients underwent LT, and the LT-free survival rate at 2 years was 90.0%. In comparison with the European reports, the patients of the present study were older and fewer underwent LT, and they seemed to have a better prognosis based on their survival rates. Our recent study of a small number of HCV-associated decompensated cirrhotic patients that compared clinical outcomes between patients who received DAA treatment and those who did not reported that liver function was improved in patients who underwent DAA treatment compared to those who did not, and mortality and incidence rates of decompensated events were lower among patients who received DAA treatment than among those who did not during an observation period of 2 years [27]. DAA treatment provides clinical benefit even for elderly decompensated cirrhotic patients or in areas where LT is not feasible.

The Japanese phase 3 study included patients with CP scores up to 12, and the prognosis after DAA treatment in patients with extremely deteriorated liver function is not clear [28]. Our study included three patients with a CP score of 12 and one patient with a CP score of 13. Among three patients with a CP score of 12, one discontinued DAA treatment due to liver failure and died on day 127 after the start of treatment. Another patient achieved SVR, but his CP score at SVR12 was 11, and he died due to HCC on day 368 after the start of treatment. The other patient achieved SVR, improved his CP score at SVR12 to 9, and did not have any decompensated event or death until the last follow-up time at 1 year after the EOT. The patient with a CP score of 13 achieved SVR, but her CP score at SVR12 remained 13, and did not have any decompensated event or death until the last follow-up time on day 547 after the start of treatment. Whether DAA treatment improves the prognosis of patients with extremely deteriorated liver function needs further study.

In the present study, the change in CP score from before to after treatment was larger than the changes in MELD score. We previously reported that improvement in albumin levels were observed in about 50% of decompensated cirrhotic patients after DAA treatment, but improvements in total bilirubin levels and prothrombin were observed in only about 10% of decompensated cirrhotic patients [6]. The differences in the before-after changes between CP class and MELD score are likely due to CP class’s inclusion of albumin level, which MELD score does not include.

The present study had some limitations. First, the number of patients who died during follow up was relatively small in this study. Second, information on diuretics, BCAA supplements, and anti-hepatic encephalopathy drugs were not collected. Third, only Japanese patients were included.

In conclusion, the prognosis of decompensated cirrhotic patients who underwent DAA treatment was clearly stratified by liver function at 12 weeks after the EOT but not baseline liver function. The absence of CP-C after DAA treatment is important in predicting a favorable prognosis for HCV patients with decompensated cirrhosis.

### Supplementary Information

Below is the link to the electronic supplementary material.

Supplementary file1 (DOCX 15 KB)

## Abbreviations

CP: Child–Pugh
HCV: Hepatitis C virus
DAA: Direct-acting antiviral
IRB: Institutional review board
SOF: Sofosbuvir
VEL: Velpatasvir
SVR: Sustained virologic response
EOT: End of treatment
MELD: Model for end-stage liver disease
INR: International normalization ratio
HE: Hepatic encephalopathy
BCAA: Branched-chain amino acid
LT: Liver transplantation
HCC: Hepatocellular carcinoma
ALT: Alanine aminotransferase
HR: Hazard ratio
CI: Confidence interval

## Declarations

### Trial registration number

University Hospital Medical Information Network (UMIN: 36150).
