# Supplementary material for: Posttreatment liver function, but not baseline liver function stratifies patient survival after direct-acting antiviral treatment in decompensated cirrhosis with hepatitis C virus
Source: J Gastroenterol. 2023 Oct 13;58(12):1211–21. doi: 10.1007/s00535-023-02039-x (PMC10657790; doi:10.1007/s00535-023-02039-x)
Supplement: Supplementary file 1 — Supplementary file1 (DOCX 15 KB) [file 535_2023_2039_MOESM1_ESM.docx]

| **Supplementary Table 1. Characteristics of patients who died or were lost to follow-up by 12 weeks after the EOT** | **Time** | **day 52** | **day 86** | **day 66** | **day 101** | **day 127** | **day 76** | **day 126** | **day 96** | **day 99** | EOT; end of treatment, MELD; model for end-stage liver disease, HT; hypertension, NEC; neuroendocrine carcinoma, DM; diabetes mellitus, Af; atrial fibrillation. |
| --- | --- | --- | --- | --- | --- | --- | --- | --- | --- | --- | --- |
|  | **Reasons leading to death or lost to follow-up** | **Death (gastric NEC)** | **Death (cancer of unknown primary)** | **Death (liver failure)** | **Death (liver failure)** | **Death (liver failure)** | **Hospital transfer** | **Personal circumstance** | **Hospital transfer** | **Personal circumstances** |  |
|  | **Comorbidity** | **HT, gastric NEC** | **DM, HT, Af** | **DM, esophageal varix** | **-** | **-** | **-** | **HT** | **HT** | **-** |  |
|  | **MELD score** | **8** | **11** | **11** | **13** | **17** | **11** | **12** | **14** | **19** |  |
|  | **Child-Pugh score** | **6** | **8** | **9** | **11** | **12** | **7** | **7** | **11** | **11** |  |
|  | **Sex** | **Male** | **Female** | **Male** | **Female** | **Female** | **Male** | **Female** | **Female** | **Female** |  |
|  | **Age**  **(years)** | **73** | **77** | **65** | **67** | **74** | **60** | **77** | **75** | **54** |  |
|  | **Case** | **1** | **2** | **3** | **4** | **5** | **6** | **7** | **8** | **9** |  |
